# Influence of Dietary Components and Traditional Chinese Medicine on Hypertension: A Potential Role for Gut Microbiota

**DOI:** 10.1155/2021/5563073

**Published:** 2021-04-20

**Authors:** Guo-Xin Zhang, Ling Jin, Hua Jin, Gui-Sen Zheng

**Affiliations:** ^1^Basic Medical College, Gansu University of Chinese Medicine, Lanzhou 730000, China; ^2^College of Pharmacy, Gansu University of Chinese Medicine, Lanzhou 730000, China; ^3^Clinical College of Chinese Medcine, Gansu University of Chinese Medicine, Lanzhou 730000, China; ^4^School of Public Health, Gansu University of Chinese Medicine, Lanzhou 730000, China

## Abstract

Hypertension (HTN) is an important worldwide public health issue affecting human health. The pathogenesis of HTN involves complex factors such as genetics, external environment, diet, and the gut microbial dysbiosis. The gut microbiota, as a medium of diet and drug metabolism, is closely correlated to host's health and disease (including HTN). Literatures were randomly collected from various databases including PubMed, ScienceDirect, Google Scholar, and China National Knowledge Infrastructure (CNKI). In this review, we elucidate the relationship between HTN and gut microbiota, as well as concerning the effects of different dietary components, diet-derived microbial metabolites, and traditional Chinese medicine (TCM) on intestinal flora. These studies have shown that diet and TCM can regulate and balance the intestinal flora, which are inclined to increasing the abundance of *Akkermansia*, *Bifidobacterium*, and *Bacteroides* and reducing the ratio of *Firmicutes* and *Bacteroidetes*. Moreover, monitoring the dynamic change of gut microflora may indicate patient prognosis and personalized response to treatment. This review aims to provide novel perspectives and potential personalized interventions for future HTN management from the perspective of gut microbiota.

## 1. Introduction

Hypertension (HTN) has become an important global public health issue because of its high morbidity and the increased risk associated with cardiovascular, stroke, and chronic kidney disease. In 2015, a total of 1.13 billion adults had HTN worldwide [1], and this number is predicted to increase to 1.56 billion by 2025 [2]. HTN creates an enormous health and economic burden if left uncontrolled, especially in low- and middle-income countries [3]. The epidemic of HTN is driven by various factors, including stress, lack of physical activity, obesity, high sodium intake, unhealthy diet, and genetic factors, which may interplay with each other and with environmental components.

The gastrointestinal tract harbors a huge community of microorganisms, which interact with the host in a symbiotic or mutualistic manner. The host provides a rich and proper nutrient habitat for the gut microflora. In reciprocation, the gut microflora and its metabolites perform various physiological functions for the host, such as maintaining the integrity of intestinal mucosal barriers, modulating energy metabolism and homeostasis, contributing the immune maturation, and resisting infection by pathogenic microorganisms [4]. A growing body of evidence have supported that environmental risk factors (including daily diet, pharmacological treatments, and pathological conditions) could influence the constitution, development, and function of the intestinal microbiota [4–10]. Among these risk factors, diet plays the dominant role in shaping the gut microbiota. One study has identified that diet changes could affect 57% of the total structural variation in gut microbiota [9]. Recent data suggest that there is a significant correlation between gut microbiota and HTN. However, little is known about the role of specific gut strains in hypertension. Moreover, monitoring of gut microflora may contribute to indicate disease manifestations, prognosis, and even personalized response to treatment.

In this review, we discuss the emerging evidence of gut microbiota and diet-derived microbial metabolites in the development and progression of HTN, as well as the influence that dietary components/TCM exert over the intestinal microbiota and HTN risk, with the aim to provide novel perspectives and potential personalized interventions for future HTN therapeutics.

## 2. Gut Microbiota and HTN

The gut microbiota is a complex and dynamic ecosystem with approximately 10^14^ microbes and mainly belongs to five phyla: *Firmicutes*, *Bacteroidetes*, *Proteobacteria*, *Actinobacteria*, and *Cerrucomicrobia*. *Firmicutes* and *Bacteroidetes* usually occupy more than 90% of the intestinal microflora [11]. The *Firmicutes/Bacteroidetes* (F/B) ratio is widely considered as a biomarker of gut dysbiosis. The development of the human microbiome seems to begin in the fetus [12–14]. The body is constantly stimulated by dietary and environmental factors after birth, and the gut microbes begin to increase and transit to a more mature pattern around 3-4 years old [15]. Over time, the microbiota of adulthood tends to be relatively stable and resilient [16, 17]. The community of microbiome is host-specific, advance, and modified throughout an individual's lifespan.

Aberrant gut microbiota was found in the onset and progression of HTN. The *Firmicutes* and *Bacteroidetes* ratio significantly increased and the gut microbial diversity and richness decreased [18]. Recent works have reported that HTN patients show significant alterations in specific bacterial groups with respect to healthy controls [19]. In particular, HTN patients exhibit a significant increase in *Prevotella, Klebsiella*, *Porphyromonas*, and *Actinomyces* and a decrease in *Bacteroides, Faecalibacterium, Oscillibacter, Roseburia, Biﬁdobacterium, Coprococcus*, and *Butyrivibrio* [19]. Moreover, there is evidence that changes in the gut microbiome also occur in prehypertensive patients, and the microbiome characteristics in prehypertension are quite similar to that in hypertension [19]. These changes might produce acetate- and butyrate-producing bacteria and accumulation of proinflammatory opportunistic pathogens, which may lead to an imbalance in intestinal microbial homeostasis (dysbiosis) that could ultimately lead to hypertension formation [18]. Santisteban and colleagues [7] also demonstrated that the microbial dysbiosis was associated with pathological changes in the gut, including increased intestinal permeability, thickened gut wall, stunted villi, and decreased number of goblet cells.

*Akkermansia muciniphila* is a mucin degrader bacteria residing in the intestinal mucus layer, which represents 3–5% of the gut microbial community. As a potential probiotic, the Gram-negative bacteria play a key role in the intestinal barrier integrity and can improve dyslipidaemia, glucose homeostasis, insulin resistance, and inflammation [20, 21]. Many studies showed that the abundance of *Akkermansia muciniphila* is decreased in obesity, diabetes, and inflammatory bowel diseases [20, 22, 23]. It is well known that obesity and diabetes are important risk factors in the incidence of hypertension. Recently, an epidemiological cohort study has demonstrated that *Akkermansia* tended to align in direction with individuals who were normotensive or had lower SBP and inversely associated with hypertension [24].

## 3. Interaction between Diet and Gut Microbiota

In fact, the bacteria eat what we eat and dietary modifications can lead to alterations in the makeup of gut microbiota. For example, infants fed with breast milk (breast-fed infants) are less diverse and have more *Bifidobacterium* in their intestines, while formula-fed infants have fewer anaerobic microorganisms with higher diversity [25, 26]. Also, differences in the gut bacterial composition were observed between African children and those living in the European urban areas [27]. The results showed that enrichment of *Bacteroidetes* and depletion of *Firmicutes* were in rural African children (diet rich in fiber, carbohydrate, and nonanimal protein), while *Enterobacteriaceae* (*Shigella* and *Escherichia*) were significantly represented in European children (high in animal protein, sugar, and fat and poor in fibers) [27, 28]. Even throughout adulthood, diet continues to be the crucial factor in modulating the microbial constitution, richness, and biodiversity [15].

Several studies have shown that long-term diet and short-term diet influence the structure and activity of the intestinal flora [29, 30]. Wu et al. [31] found that prevalence of *Bacteroides* is highly associated with high-fat and animal protein intake, whereas high *Prevotella* is with complex carbohydrate-based intake. Although the detectable change of microbiome composition occurred within 24 hours, the enterotype remained stable during the 10-day study, suggesting that the manipulation of the gut microbiota via dietary patterns should be long term. In addition, the gut microbial communities can also rapidly and consistently respond to short-time dietary change [32, 33]. For example, lack of fiber in an animal-based diet increased the abundance of bile-tolerant microorganisms (*Alistipes*, *Bilophila*, and *Bacteroides*) and decreased the levels of *Firmicutes* that metabolize dietary plant polysaccharides (*Roseburia*, *Eubacterium rectale*, and *Ruminococcus bromii*) [29].

Dietary nutrient exchange is the bond that maintains the symbiotic nature of host-gut microbes. The host and the intestinal microbes would coproduce a myriad of small-molecule bioactive metabolites during the metabolic transformation of dietary compounds. On the one hand, many of small molecules affect information exchange between host cells and the host's microbial symbionts. On the other hand, some of the transformations would selectively improve the biological activity of their products and would obtain partially lost energy from the host. Due to its high abundance and diversity, the gut microbiota is particularly enriched in genes that encode various enzymes essential for metabolizing several macronutrients (especially dietary complex polysaccharides), which reciprocally modulate the composition, gene expression, and metabolism of the gut microbiota [34].

For example, herbivorous microbiomes were enriched in enzymes that map to biosynthetic reactions for hydrolysis of plant polysaccharides and amino acids building blocks, whereas carnivorous communities were enriched in enzymes to degrade proteins as an energy source [35]. Thus, diet and gut microbiota are considered to have a complex bidirectional relationship, whereby the diet influence the microbiota, and gut microbiota reciprocally allow fermentation and biotransformation of dietary ingredients in response to dietary nutrient availability.

## 4. Dietary Intervention in HTN

Diet is the most modifiable component of lifestyle that can modulate the onset and development of hypertension. For example, the Dietary Approaches to Stop Hypertension (DASH) diet is a widely recommended diet for individuals with high blood pressure, which is rich in vegetables, fruits, low-fat dairy, and whole grains and low in total and saturated fat. Many research studies showed that dietary intervention (fiber, polyphenols, and herbal components) is a safe and effective strategy for prevention and treatment of HTN, which may be linked to improved gut health and degraded microbial metabolites.

### 4.1. Dietary Fiber

Dietary fiber is a nondigestible form of carbohydrates. Based on their water solubility, dietary fiber is classified as soluble and insoluble [36]. Soluble fiber is viscous, readily enabling the gut contents to form a gel-like consistency that delays emptying of the gut and also fermentable in the colon by promoting the growth of intestinal and fecal microbiota and their by-products [37]. On the other hand, insoluble fiber is nonviscous which may decrease the intestinal transit time and increase fecal bulking by the nature of the particle formation and water holding capacity, thus promoting digestive regularity [38].

#### 4.1.1. Epidemiological Associations between Dietary Fiber and HTN

A randomized double-blind trial reported that a diet rich in fiber reduced significantly the systolic and diastolic blood pressure by 1.8 mmHg and by 1.2 mmHg, respectively, compared with placebo following 12 weeks [39]. Another randomized, controlled, parallel-group pilot study observed that 5.52 g/day oat *β*-glucan induced a 7.5 mm Hg reduction in systolic blood pressure (SBP) and a 5.5 mm Hg in diastolic blood pressure (DBP) for 6 weeks in 18 untreated hypertensive patients, suggesting that whole oats rich in soluble fiber may be an effective dietary therapy [40]. Similarly, daily coarse food grain (coarse food grains are rich in fiber, including grains or beans other than rice and wheat products) intake and the frequency of coarse food grain intake were inversely associated with both SBP and DBP among young Chinese adults [41]. More recently, Aljuraiban et al. [42] investigated cross-sectional associations with BP of total, insoluble and soluble fiber intake among the 2195 participants aged 40–59 years from the USA. The finding indicated that a higher intake of total dietary fiber, especially insoluble was associated with lower SBP, but not with soluble fiber. Similar results were also observed in other studies [43, 44]. Taken together, a relatively high intake of dietary fiber was inversely associated with the risk of hypertension, but the effect of different dietary fiber types (soluble and insoluble dietary) on BP was inconsistent, and the results may be related to study design and methodology or difference in soluble and insoluble dietary fiber intakes.

#### 4.1.2. Antihypertension Mechanism of Dietary Fiber

With increasing economic development, the increased consumption of energy-dense and processed foods, and reduced consumption of dietary fiber have been considered the leading risk factor for HTN. Furthermore, gut microbial dysbiosis related to HTN pathology was involved in nutrient metabolism, systemic immunity, inflammation, and the bowel function of the gut barrier [45, 46].

Dietary fiber ingestion reduced the *Firmicutes* to *Bacteroidetes* ratio, decreased *Enterobacteriaceae* and *Prevotella genus*, and increased abundance of some species (such as *Bifidobacterium, Lactobacillus,* and *Bacteroides*) [47–49], which effectively improves the gut mucus layer function, reduces bacterial translocation and infection, and constructs a healthier intestinal ecosystem [50, 51].

Short-chain fatty acids (SCFAs), end products of the microbial fermentation by the gut (mostly acetate, propionate, and butyrate), are primarily derived from dietary fibers. SCFAs can bind and activate host receptors and act as a “communication” pathway between the gut microbial metabolism and host physiology. Of note, SCFAs can directly activate specific distinct G-protein-coupled receptors (including G-protein receptor 41 (Gpr41), G-protein receptor 43 (Gpr43), and olfactory receptor78 (Olfr78)) and regulate BP [52, 53]. Olfr78 is coupled with SCFAs to induce renin release and increase blood pressure, in which this process can be regulated by Gpr43-mediated vasodilation. SCFAs receptors, Olfr78 and Gpr41, are expressed in smooth muscle cells of small resistance vessels, and they are mutually antagonistic, such as Olfr78 knockout mice that are hypotensive, whereas Gpr41 knockout mice that are hypertensive [53]. In addition, SCFAs play diverse roles such as providing energy requirements for cell growth and differentiation, adding the gut microbial diversity, anti-inflammation, and immunomodulatory, promoting gut epithelial integrity, and regulating lipid, glucose, and cholesterol metabolism [54, 55]. Above all, these data have shown that SCFAs can directly and indirectly affect BP.

### 4.2. Dietary Fat

Increased fat intake induces gut dysbiosis and increases the risk of HTN [56]. Consumption of high-fat (HF) diet was found to be associated with the shift of gut microbiota and inhibit nutrient-sensing signals in hypertensive animals. In the study of maternal and postweaning HF diet-induced hypertension in adult male offspring, HF diet increased the F/B ratio and the abundance of genus *Clostridium* and decreased the abundance of genus *Lactobacillus* and *Turicibacter*, as well as inhibited the AMPK–PGC-1*α* signal pathway. Notably, maternal HF diet reduced the abundance of genus *Akkermansia*, but the opposite was true after postweaning [57]. Similar results were obtained by Chen et al. [58]. Wang et al. [59] showed that low-fat diet can increase the gut microbial *α*-diversity and the abundance of *Blautia* and *Faecalibacterium*, while decreasing the cometabolites p-cresol and indole (amino acid metabolites associated with host metabolic disorders). The HF diet intake can reduce the enrichment of *Bacteroides*, *Alistipes*, and *Faecalibacterium* as opposed to the low-fat diet group. In addition to the unbalance of gut microbiota, the HF diet reduced the concentrations of fecal butyrate acid and SCFAs and increased plasma proinflammatory factors [59].

The type of fat consumed also plays an important role for BP. Dietary fat can be broadly broken down into three broad subtypes—SFA (saturated fatty acids), UFA (unsaturated fatty acids), and transfat. A prospective urban rural epidemiology (PURE) study found that higher intakes of total fat and saturated fat acids were positively correlated with BP, whereas replacement of saturated fatty acids with unsaturated fats resulted in a decrease in SBP and DBP [60]. Wolters et al. [61] found a significant difference among observational studies. Further analysis by the type of fat showed that microbiota richness and diversity was negatively correlated with high intake of fat and SFA. SFA promoted the enrichment of *Clostridium bolteae* and *Blautia*, which in turn induced insulin resistance and increased BMI. High dietary monounsaturated fatty acids (MUFA) may decrease total bacterial numbers, while poly-unsaturated fatty acids (PUFA) had no effect on the richness and diversity of gut microbiota [61]. Mice supplemented with dietary docosahexaenoic acid (DHA) for 12 weeks had improved left ventricular function, reduced wall shear stress, and oscillatory shear at ostia in the descending aorta, and significantly lowered blood pressure as well [62].

### 4.3. Trimethylamine-N-Oxide (TMAO): Gut Microbial Metabolite

Trimethylamine-N-oxide (TMAO) is a gut microbial metabolite. Dietary choline, carnitine, and betaine are metabolized by gut microbial enzymes to generate trimethylamine (TMA) and then absorbed by the gut and delivered to the liver where it is metabolized by flavin monooxygenases (FMOs) to form TMAO.

The meta-analysis of the relation between circulating TMAO concentration and HTN prevalence in a large population (11,750 individuals and 6176 hypertensive cases) demonstrated that a significant positive dose-dependent association between circulating TMAO concentrations and hypertension risk [63]. Specifically, the HTN risk could increase by 9% per 5 *μ*mol/L and 20% per 10 *μ*mol/L increment of circulating TMAO concentration. Patients in high circulating TMAO levels had a 12% increased risk of HTN compared with those in the low circulating TMAO levels [63].

In another study, Nie and collaborators [64] conducted a nested case-control study (including 622 patients with first stroke and 622 matched controls), in which elevated TMAO levels were significantly associated with increased risk of first stroke in hypertensive patients over a 4.5-year follow-up period, and hemorrhagic stroke was more common than ischemic stroke. It is known that HTN is one of the most important risk factors for stroke. Furthermore confirmed, baseline folate levels modified the effect of TMAO on the incidence of first stroke and first ischemic stroke. High folate and low TMAO had the lowest rate of stroke, whereas low folate and high TMAO had the highest rate of stroke [64]. Authors should discuss the results and how they can be interpreted in perspective of previous studies and of the working hypotheses. The findings and their implications should be discussed in the broadest context possible. Future research directions may also be highlighted.

TMAO levels have been found to correlate with human gut microbial enterotypes. Higher plasma concentrations of TMAO were found to be associated with the genus *Prevotella* enterotype, as opposed to the genus *Bacteroides* enterotype [65]. High-TMAO producers (≥20% increase in urinary TMAO in response to eggs and beef) had 58.1% *Firmicutes* to 32.6% *Bacteroidetes* (∼2 : 1 *Firmicutes* : *Bacteroidetes*), whereas low-TMAO producers (<20% increase in urinary TMAO) had 47.7% *Firmicutes* to 47.2% *Bacteroidetes* (1 : 1 *Firmicutes* : *Bacteroidetes*). Moreover, high-TMAO producers had lower *α*-diversity (within-individual) measure than low-TMAO producers [66]. High-salt diet (HSD) is a recognized important risk factor for HTN. In an animal model study, high salt intake increased the plasma TMAO level, whereas it decreased 24-hour TMAO urine excretion and affected the overall composition of gut bacteria in rats as well [67]. Obstructive sleep apnea (OSA) has been labeled as an important identifiable cause for the development of hypertension. Liu et al. [68] modeled OSA-induced HTN in rats and found that OSA synergized HSD to increase the severity of HTN though increasing the level of blood TMAO. Treatment using *Lactobacillus rhamnosus* GG strain reversed the above findings.

However, there are conflicting data on whether TMAO is beneficial or harmful for a living organism. Several biophysical studies showed that TMAO exerts a protective effect, such as stabilization of proteins and nucleic acids, protecting cells from osmotic and hydrostatic pressure stresses [69]. Recently, we found that beyond increasing plasma TMAO by 4-5 fold, a chronic, low-dose TMAO treatment also reduced vasopressin and cardiac fibrosis and improved hemodynamic and biochemical parameters of failing heart in spontaneously hypertensive rats [70]. Therefore, further studies are needed to fully assess the effect of TMAO on the lifestyle diseases.

### 4.4. Polyphenols

Polyphenols are a large group of natural compounds with high chemical diversity that are synthesized by plants, which are generally defined as dietary antioxidants. Recently, dietary polyphenols is also proposed as potential prebiotics which can affect the gut microbiota diversity and composition [71, 72]. Once ingested, polyphenols are generally poorly absorbed in the small intestine, mostly into the colon where they are modified by the resident microbiota to be degraded into smaller metabolites such as phenolic acids to increase its biological activities for the host utilization [73, 74].

Numerous studies indicated that dietary polyphenols might reduce the cardiovascular risk and metabolic disorders [75, 76], such as lowering of blood pressure, modulating blood glucose, improvement of endothelial function, and reducing plasma lipids [77]. The main antihypertension mechanisms of dietary polyphenols include anti-inflammation, amelioration in endothelial function via the NO-cGMP pathway and angiotensin-converting enzyme (ACE) inhibition [78], and the regulation of oxidase and antioxidant enzyme genes expression to prevent oxidative stress had been reviewed previously [79–81]. In fact, polyphenols were also found to play a role in reshaping the gut microbial community to provide beneficial effects on blood press regulation [82]. Resveratrol (RV), a polyphenolic component of grape and red wine, has been shown to improve vascular function and attenuate high blood pressure [83, 84]. Recently, many studies have documented that resveratrol could regulate the gut microbiota composition and diversity. Particularly, resveratrol decreases the F/B ratios, increases the abundances of *Lactobacillus*, *Bifidobacterium*, and *Akkermansia*, and inhibits the growth of *Enterococcus faecalis* [85, 86]. Furthermore, studies suggest that resveratrol could also promote the population of butyrate producer *Blautia* and *Dorea* in the *Lachnospiraceae* family and increase SCFAs production [87, 88].

In addition, resveratrol can reduce TMAO levels by remodeling intestinal flora, thereby preventing TMAO-induced atherosclerosis in ApoE/mice. Obesity is closely related to HTN. In the cafeteria diet-induced obesity experiment, hesperidin supplementation (a flavanone glycoside mainly present in citrus fruits) significantly reduced systolic blood pressure in obese rats by altering microbiota diversity [82].

## 5. Traditional Chinese Medicine

Traditional Chinese medicine (TCM) is also known as botanical medicine or phytomedicine; one important advantage of TCM is that patients are treated holistically through multiple targets. Numerous research studies on TCM management of hypertension and its complications have been published. Oral TCM decoction is the main way of administration. The herb travels to the gut and comes in contact directly with the gut flora. Then, the bacterial enzymes in the gut will convert the herb into active ingredients. Recent studies have shown that TCM monomers and formulae might improve the symptoms of hypertension by modulating the gut microbiota (Table 1).

### 5.1. Berberine

Berberine (BBR), an isoquinoline alkaloid extracted from many medicinal herbs (including *Coptis* root and *Phellodendron chinense*), has the clinical effect of clearing “heat,” purging “fire,” and Alevi pharmacons. A growing body of evidence from both animal and clinical investigations has shown that BBR and its derivatives have the effect of lowing blood pressure in patients with hypertension, and its mechanism involved that inhibit RAS activity and insulin resistance, decrease levels of aldosterone [93, 94], reduce arterial stiffness [95], and improve endothelial function [93].

Recent studies have shown that BBR can also play an effective role by influencing the gut flora. In the experiment of atherosclerosis induced by HF diet in mice, Zhu et al. [96] reported that BBR treatment increased the relate abundance of *Akkermansia* and *Bacteroides* in the gut, improved the number of goblet cells, restores the thickness of the colonic mucus layer, reduced metabolic endotoxemia and systemic inflammation, and then significantly ameliorated atherosclerosis. BBR can induce better glycometabolism through regulating the gut microbiota and fecal metabolomics in type-2 diabetic db/db mice, which is specifically manifested by decreased abundance of *Saccharibacteria*, *Deferribacteraceae*, *Actinobacteria*, and *Firmicutes* and increased abundance of *Verrucomicrobia* [97]. At present, only a limited number of studies examining the role of BBR through gut microbiota in HTN have been performed. Wu et al. [89] indicated that Sanoshashinto (SHXXTM) and the berberine–baicalin (BB) combination reduced left ventricular hypertrophy and altered gut microbiota (enhancing the amount of *Lactobacillus*), furthermore to reduce HTN. BBR and baicalin were the main antihypertensive constituents in Sanoshashinto. Therefore, further in vitro and in vivo experiments are needed to verify.

### 5.2. Baicalin

Baicalin is a flavonoid component extracted from the dried root of *Scutellaria baicalensis* Georgi that has significant anti-inflammatory, antioxidative, and antihypertensive activities, so baicalin is widely used to treat HTN, cardiovascular diseases, and inflammatory diseases [98, 99].

The effect of baicalin on gut microbiota and mucosal immunity has been reported in the spontaneously hypertensive rats. The result showed that baicalin increased the amount of SCFAs, including acetic acid, propionic acid, butyric acid, isobutyric acid, valeric acid, and isovaleric acid. Through 16S rDNA sequencing of the fecal samples, the genus *Roseburia*, *Akkermansia*, *Allobaculum*, *Bifidobacterium*, *Ruminococcaceae*, and *Ruminococcus* 2 were increased after baicalin treatment, which are SCFAs-producing bacteria [90].

Baicalin treatment can increase the ileal and colonic expression of zonula occluden-1 (ZO-1) and cingulin occluding, which maintain intestinal tight junction and regulate the permeability of the intestinal epithelial barrier [90]. Baicalin treatment also decrease Tlr2, IL-1*β*, TNF-*α*, and IL-23 in the spontaneous hypertension rats (SHRs) and restrict the intestinal inflammatory response. In addition, baicalin can also attenuate impairment of the mechanical intestinal barrier and intestinal fibrotic lesions [90].

### 5.3. The Combination of *Astragalus membranaceus* and *Salvia miltiorrhiza* (HD)

HuangQi (*Astragalus membranaceus*) possesses nourishing qi, activating blood circulation, tonic, hepatoprotective, and diuretic properties. It has been proven to be beneficial to immunoregulation, anti-inflammation, insulin resistance, and BP control. Danshen (*Salvia miltiorrhiza*) is a member of herbal medicine, which has the functions of promoting blood circulation, removing blood stasis, clearing away heart fire, and tranquilizing the mind. It has been proven to be beneﬁcial to inducing vasodilation [100], ACE inhibition [101], antithrombosis [102], cardioprotection [103], and antihypertension [100, 101] in animal models and human studies. *Astragalus membranaceus* and *Salvia miltiorrhiza* have long been used in TCM and serve as the principal herbs in treating HTN. Previous studies had found that HD had a better antihypertensive effect than the separated use of herb [91]. The network pharmacology approach revealed that *Astragalus* and *Salvia* compound can attenuate impairment of endothelial cell, promote placental trophoblast cells and vascular endothelial cells synthesizing, and then prevent the development of pregnancy-induced HTN syndrome [104].

HD improved the structure and composition of imbalance intestinal flora. It was reported that HD treatment reduced the ratio of F/B in the intestinal microflora of SHRs, while the relative abundance of *Akkermansia*, *Akkermansia muciniphila*, and *Lactobacillus intestinalis* was signiﬁcantly increased in HD-treated rats than in SHRs. The correlation analysis demonstrated that HD also changed the serum metabolic pattern of SHRs, which mainly enriched in arachidonic acid metabolism, glutathione metabolism, steroid hormone biosynthesis, and tryptophan metabolism and play a role in anti-inflammatory, antiplatelet aggregation, vasodilatation, and antioxidative stress effects [91].

### 5.4. Zhen Gan Xi Feng Decoction (ZGXFD)

ZGXFD is a Chinese herbal formula recorded in the book “Medical Zhong parameter West recorded” written by Xichun Zhang. ZGXFD consists of extracts of *Achyranthes* root, ruddle, dragon bone, oyster shell, plastrum testudinis, white peony root, Radix Scrophulariae, Radix Asparagi, Fructus Toosendan, raw malt, *Artemisia capillaris* thunb, and *Glycyrrhiza*. In some clinical observations and animal trial, ZGXFD has been reported to have beneficial effects on HTN. For example, ZGXFD regulates gastrointestinal hormones, blocks the rennin-angiotensin-aldosterone system, depresses sympathetic nerves, improves insulin resistance, inhibits the apoptosis of vascular smooth muscle cells, and plays a good role in lowering blood pressure. Meta-analysis also showed that ZGXFD may be more effective in BP control and improving clinical symptoms and signs such as dizziness, headache, tinnitus, palpitations, insomnia, and irritability than antihypertensive drugs [105, 106].

Yu et al. [92] have addressed the effect of ZGXFD on gut microbiota diversity and composition in SHRs. After 8 weeks of treatment with ZGXFD, the gut microbial diversity, F/B ratio, and *coccus* to *bacillus* (C/B) ratio were decreased. *C/B* ratio is also commonly used as an indicator of gut bacterial homeostasis. ZGXFD increased the fecal SCFAs production via gut microbial composition remodeling, thereby maintaining the integrity of the mucosal barriers. Furthermore, D-lactic acid and DAO in blood, a marker for the integrity of intestinal barrier, were decreased in the ZGXFD-treat group when compared with the SHRs group. Although limited, available information has demonstrated that ZGXFD is beneﬁcial to gut microbial homeostasis and integrity of intestinal mechanical barrier and thus exerts an antihypertensive effect [92].

### 5.5. Sanoshashinto (San Huang Xie Xin Tang in China, SHXXTM)

SHXXTM is a classical prescription of ancient Chinese medicine, with the earliest record being found in the book “Synopsis of The Golden Chamber” compiled by Zhongjing Zhang. SHXXTM consists of Dahuang (Rhei Rhizoma), HuangQin (Scutellariae Radix), and Huang Lian (Coptidis Rhizoma). It has been used clinically to treat acute ischemic stroke, HTN, and hyperlipidemia [107–109]. Studies showed that SHXXTM decrease the level of inflammatory factors, clear away heat and toxic materials, regulate gut flora through purgation, reduce the production of TMAO, and thus improve stroke and prognosis [107]. In addition, SHXXTM relaxes blood vessels [109], lowers serum cholesterol [108], increases the insulin sensitivity index [110] and antioxidant activities [111], and plays a positive role in the treatment of HTN.

A recent study showed that treatment with SHXXTM (800 mg/kg/d) for 6 weeks alter the percentage compositions of *Corynebacterium*, *Lactobacillus*, and *Turicibacter* bacteria. SHXXTM treatment could increase the amount of *Lactobacillus* and then ameliorate BP [89]. In addition, SHXXTM reduced left ventricular hypertrophy and vessel wall thickness. Thereby, this provides a new provement for the antihypertensive mechanism of SHXXTM.

## 6. Conclusions

Undoubtedly, the gut microbiota has gained growing attention as a novel therapeutic target in the management of hypertension. In this review, we described some important and effective dietary components and TCM that improve HTN by regulating the intestinal microbiota. The use of these materials (dietary fiber, polyphenols, and TCM) could exert prebiotics-like activities, which are tended to increase the abundance of *Akkermansia*, *Lactobacillus*, *Bifidobacterium,* and *Bacteroides* and decreasing *Firmicutes* /*Bacteroidetes* ratio to rebalance the dysbiotic hypertension gut microbiota, so dietary and TCM intervention could be a promising and important approach in controlling HTN. However, the available data in this ﬁeld are still limited. Further investigations are needed to clarify how gut microbiota alters blood pressure and vascular function and which specific gut microbial strains contribute to HTN development. In addition, the plasticity and interindividual differences of intestinal microbiomes are also important factors to be considered in the treatment or prevention of disease. We speculate that in the future, intestinal flora targeted therapy by Chinese herbs will be dedicated to precise and individualized optimization. Moreover, the safety and security of Chinese herbs should be also analyzed before using. Therefore, we need to provide more scientific evidence and clinical validation of these underlying mechanisms to pave the way for individualized intervention strategies for hypertension.

## Figures and Tables

**Table 1:** Changes in microbiota composition related to HTN and Chinese herbal products.

Category	Chinese herbal products	Method	Implicated microbiota	Reference
Monomers	Berberine	16s rDNA	*Lactobacillus* ↑	Wu et al., 2020 [89]
	Baicalin	16s rDNA	*Roseburia* ↑ and *Akkermansia* ↑	Wu et al., 2019 [90]
			*Allobaculum* ↑ and *Bifidobacterium* ↑	
			*Ruminococcaceae* ↑ and *Ruminococcus* 2↑	
Formulae	HD	16s rDNA	*Firmicutes*/*Bacteroidetes* (F/B) ↓	Han et al., 2019 [91]
			*Akkermansia* ↑ and *Akkermansia muciniphila* ↑	
			*Lactobacillus intestinalis* ↑	
	ZGXFD	16s rDNA	*Firmicutes*/*Bacteroidetes* (F/B) ↓	Yu et al., 2019 [92]
	SHXXTM	16s rDNA	*Coccus*/*Bacillus* (C/B) ↓	Wu et al., 2020 [89]
			*Lactobacillus* ↑ and *Turicibacter* ↑	
			*Corynebacterium* ↓	

## Data Availability

This study is a review article, and no data were used to support this study.
